# Aseptic Meningitis as an Initial Presentation of Primary Sjögren's Syndrome: A Case Report

**DOI:** 10.7759/cureus.71252

**Published:** 2024-10-11

**Authors:** Sara Alnajjar, Jumanah Alfuwayris, Abdulmajid M Al Arfaj, Abdulmohsen Almukhaitah, Munirah Almakhayitah, Naimah A Al-Naim, Reem Alharshan

**Affiliations:** 1 Neurology, King Abdulaziz Hospital, Al Ahsa, SAU; 2 Rheumatology, King Abdulaziz Hospital, Al Ahsa, SAU; 3 Infectious Diseases, King Abdulaziz Hospital, Al Ahsa, SAU; 4 Internal Medicine, King Abdulaziz Hospital, Al Ahsa, SAU

**Keywords:** aseptic meningitis, autoimmune diseases, dry eye and mouth, headache, primary sjogren’s syndrome, sjögren's syndrome

## Abstract

Neurological symptoms that occur before the diagnosis of primary Sjögren's syndrome (PSS) can vary and affect either the central nervous system (CNS) or the peripheral nervous system (PNS). Aseptic meningitis, although rare, can be an initial central neurological sign of PSS. This case report describes a 54-year-old patient who was initially presented with aseptic meningitis and was subsequently diagnosed with PSS. The diagnosis was based on clinical features and the results of serum autoantibody tests. The patient did not undergo a minor salivary gland biopsy to confirm the diagnosis. Symptomatic management resulted in an improvement in the patient’s condition.

## Introduction

Aseptic meningitis in primary Sjögren's syndrome (PSS) is a rare presentation that has been previously described [1,2]. PSS is an autoimmune exocrinopathy characterized by inflammatory lymphocytic infiltration of the exocrine glands, primarily the salivary and lacrimal glands, with variable manifestations affecting multiple organ systems [1,2]. The neurologic manifestations of PSS can be classified anatomically into peripheral nervous system (PNS) and central nervous system (CNS) conditions [3]. CNS involvement is observed in approximately 14% to 19% of cases [1].

Here, we report a patient with aseptic meningitis who tested positive for anti-Ro/SSA and anti-La/SSB antibodies.

## Case presentation

**Figure 1:**
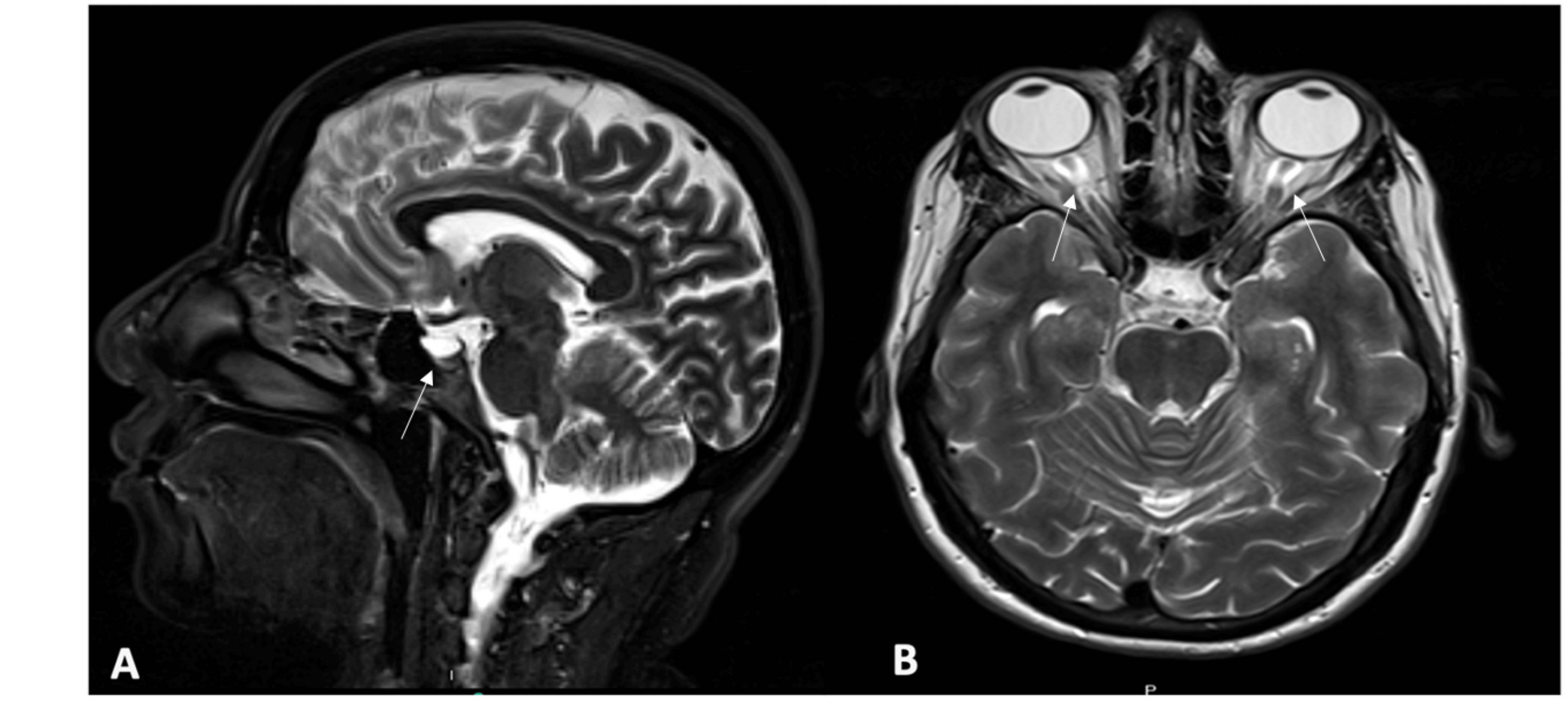
Brain magnetic resonance imaging. Brain MRI T2 fluid-attenuated inversion recovery sequence sagittal (A) and axial (B) views showed partially empty sella turcica and prominent subarachnoid space around the optic nerve.

**Figure 2:**
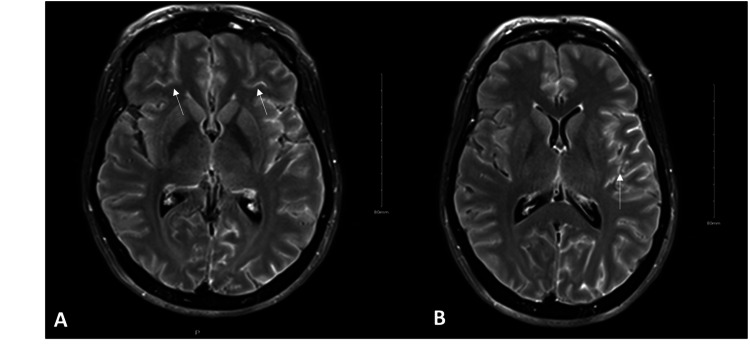
Brain magnetic resonance imaging. Brain MRI T2 fluid-attenuated inversion recovery sequence on gadolinium-enhanced axial views (A and B) revealed leptomeningeal enhancement along the bifrontal lobes and the left Sylvian fissure.

**Figure 3:**
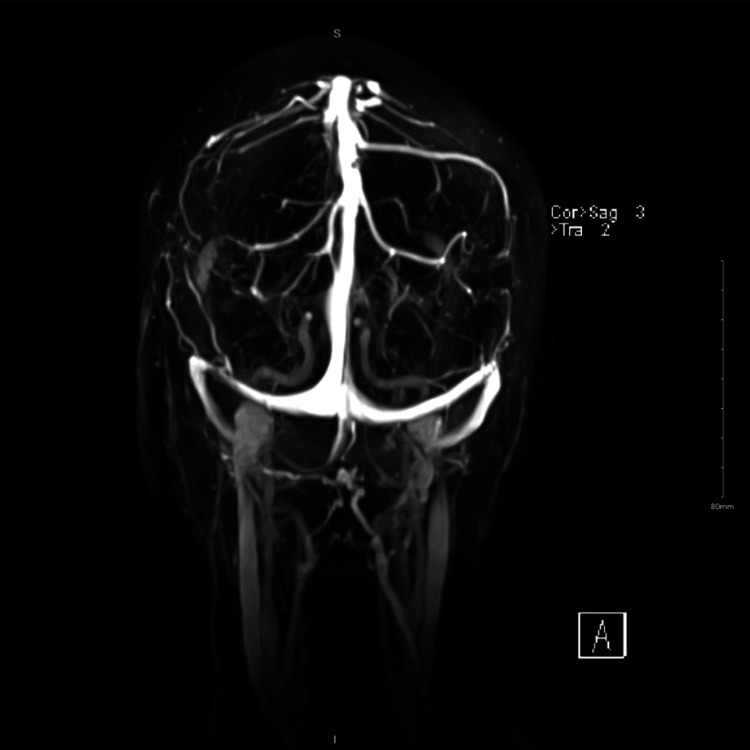
Brain magnetic resonance venography was unremarkable.

A 54-year-old female experienced a gradual onset of headache, along with pain when moving her eyes, vomiting, and a documented fever of 38.2°C. She reported a history of mild numbness in both feet, as well as excessive thirst. Prior to this episode, the patient had no known medical condition and denied using any medications in the six months leading up to her presentation. Neurological examination, including fundoscopy, was normal. A brain magnetic resonance imaging (MRI) with and without contrast revealed leptomeningeal enhancement in the bilateral frontal lobes and left Sylvian fissure (Figures 1, 2). Brain magnetic resonance venography (MRV) showed no abnormalities (Figure 3). Cerebrospinal fluid (CSF) analysis showed a protein level of 1.75g/L, white blood cell count (WBC) of 45 x 10^6/L, red blood cell count (RBC) of 98 x 10^6/L, and a glucose level of 3.1 mmol/L. The opening pressure was 23 mmHg. CSF culture and meningitis multiplex panel were negative. A diagnosis of aseptic meningitis was made, and the patient was started on empirical therapy with intravenous (IV) ceftriaxone, vancomycin, and acyclovir. This treatment was discontinued after completing the course. The patient was kept on IV fluids and given naproxen 500 mg as needed.

Further investigations were conducted and revealed an anti-nuclear antibodies (ANA) level of 156.863 units, an anti-Ro/SSA level of 101.925 units, an anti-La/SSB antibody level of 21.292 units, erythrocyte sedimentation rate (ESR) of 60 mm/hr, and C-reactive protein (CRP) of 33.4 mg/L. Screening for other autoimmune diseases yielded negative results. A nerve conduction study (NCS) showed normal results. The patient declined to undergo a salivary gland biopsy and was reluctant to start hydroxychloroquine. Her initial symptoms, including headache, resolved after completing the antibiotics course, and she continued to be closely monitored in the clinic.

## Discussion

The diagnosis of aseptic meningitis secondary to PSS was determined based on the clinical data and investigation results in our case. It was confirmed that there was no presence of another autoimmune disease. The prevalence of neurologic involvement in Sjögren's syndrome (SS) varies widely due to referral bias, different diagnostic criteria, variations in seeking neurologic manifestations, and whether these manifestations are clinically overt or asymptomatic [4,5].

Approximately 20% of patients with PSS can have CNS involvement, which can lead to acute, sometimes recurrent, episodes with varying outcomes [4]. The first reported case of recurrent aseptic meningoencephalitis as a neurological manifestation of PSS was documented by Alexander and Alexander in 1983 [6]. The exact etiology of aseptic meningitis in SS is not yet fully understood [5]. Furthermore, prior literature indicates that the underlying pathogenesis of CNS involvement in PSS may be primarily due to immunologically mediated small vessel vasculopathy, with small vessel vasculitis being less common [4].

Given her young age and the lack of risk factors, we explored potential immunological and infectious causes of meningitis. After careful evaluation, we diagnosed our patient with aseptic meningitis and PSS. This diagnosis was supported by positive ANA, anti-Ro/SSA, and anti-La/SSB antibodies, as well as clinical symptoms of dry eye and mouth. We ruled out other potential triggers of aseptic meningitis, including drugs, bacteria, and viruses.

Anti-Ro/SSA antibodies are known to be associated with CNS vasculitis secondary to SS, which explains the pathophysiology of meningitis [7]. Necrotizing vasculitis involving the entire neuroaxis has been observed in two autopsied cases of SS, and T- and B-lymphocytic perivascular leptomeningeal and intraparenchymal infiltration were described [8]. There is a lack of published case reports on aseptic meningitis secondary to SS [1,9,10].

## Conclusions

In summary, this case presents aseptic meningitis as the initial manifestation of PSS, which was successfully managed symptomatically. SS should be considered in the differential diagnosis of recurrent aseptic meningitis.
